# Supplementary figures and images for: Septin7 is indispensable for proper skeletal muscle architecture and function
Source: eLife. 2022 Aug 5;11:e75863. doi: 10.7554/eLife.75863 (PMC9355566; doi:10.7554/eLife.75863)

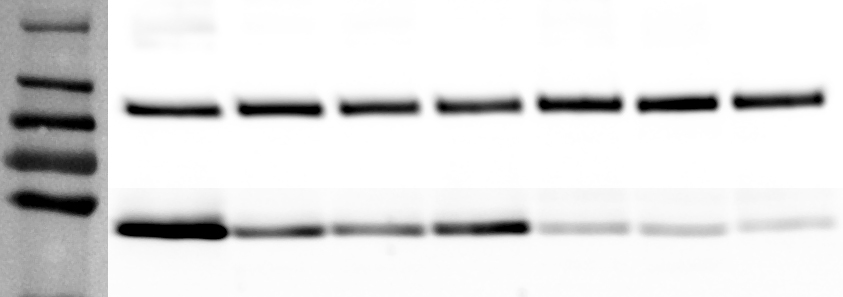

Supplement: Figure 1—source data 7. [file elife-75863-fig1-data7.jpg]

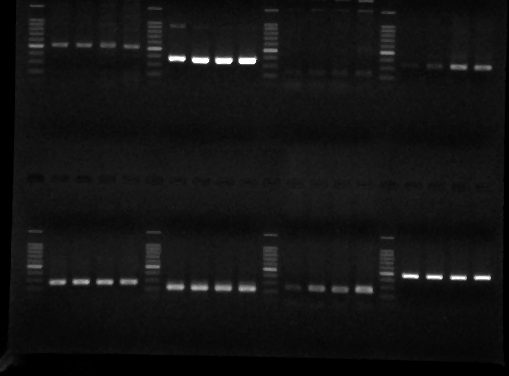

Supplement: Figure 1—figure supplement 1—source data 4. — Proliferating cells (p1) and myotubes at different stages of development (d1, d3, and d5) are demonstrated. Relevant parts of the original gel images are presented in Figure 1—figure supplement 1B. [file elife-75863-fig1-figsupp1-data4.tif]

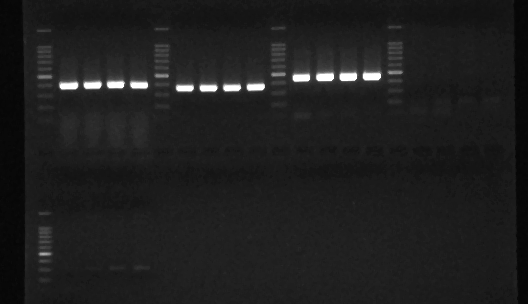

Supplement: Figure 1—figure supplement 1—source data 5. [file elife-75863-fig1-figsupp1-data5.tif]

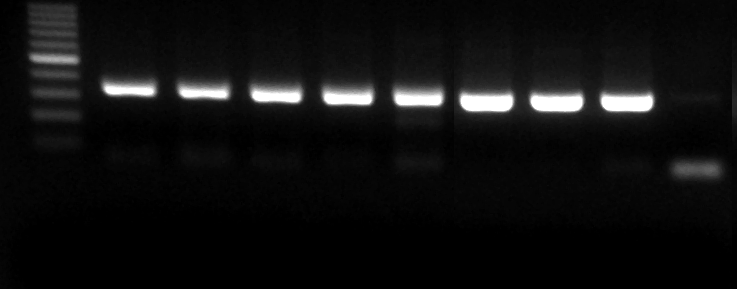

Supplement: Figure 1—figure supplement 1—source data 6. — Gapdh was used as loading control; NB: newborn muscle; TA: m. tibialis anterior; FDB: m. flexor digitorum brevis; EDL: m. extensor digitorum longus; and Sol: m. soleus were examined. Relevant parts of the original gel images are presented in Figure 1—figure supplement 1C. [file elife-75863-fig1-figsupp1-data6.tif]

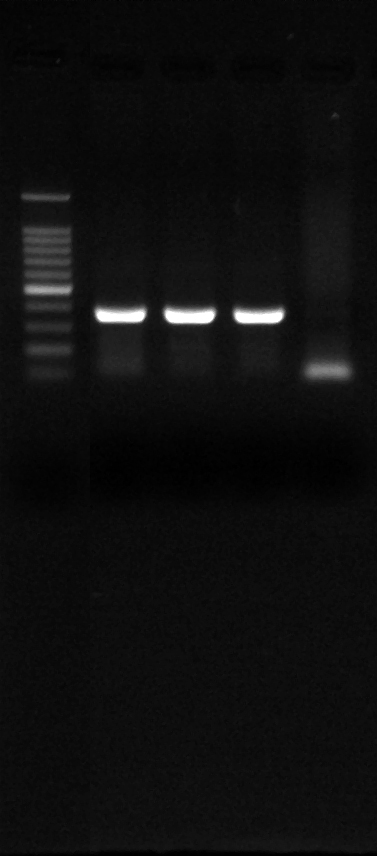

Supplement: Figure 1—figure supplement 1—source data 7. [file elife-75863-fig1-figsupp1-data7.jpg]

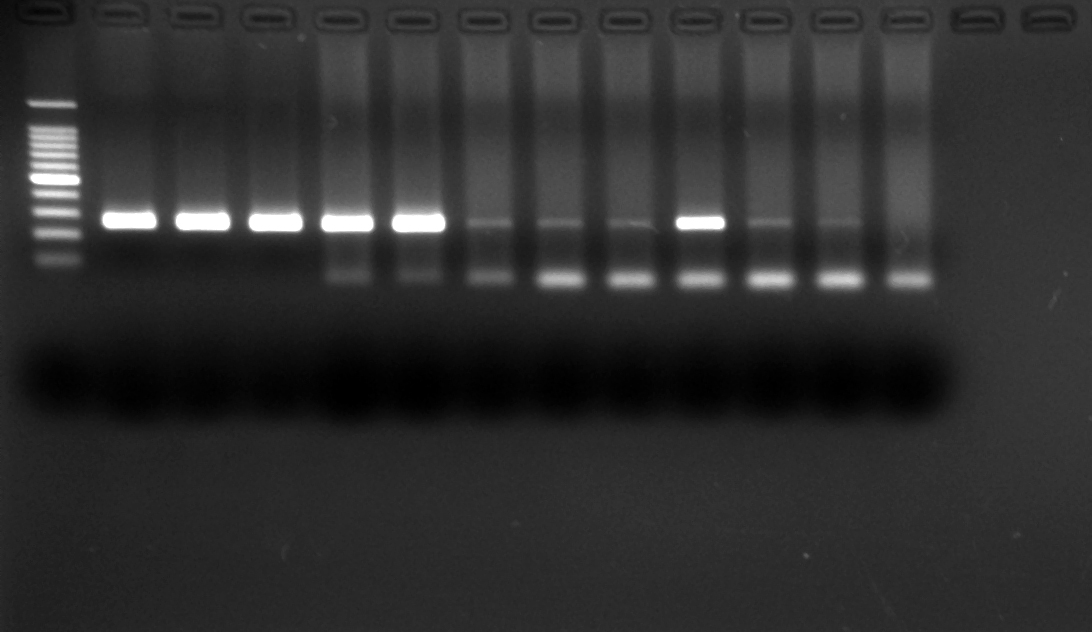

Supplement: Figure 1—figure supplement 1—source data 8. [file elife-75863-fig1-figsupp1-data8.jpg]

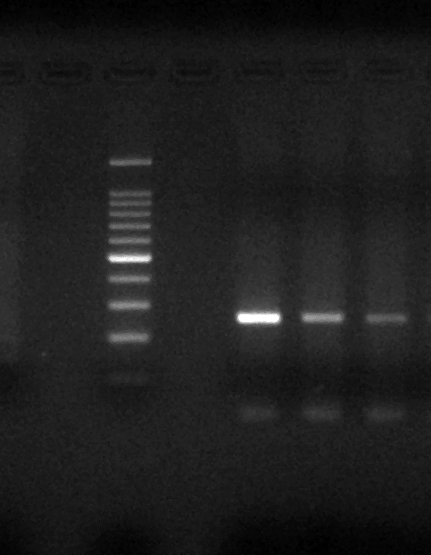

Supplement: Figure 1—figure supplement 1—source data 9. [file elife-75863-fig1-figsupp1-data9.jpg]

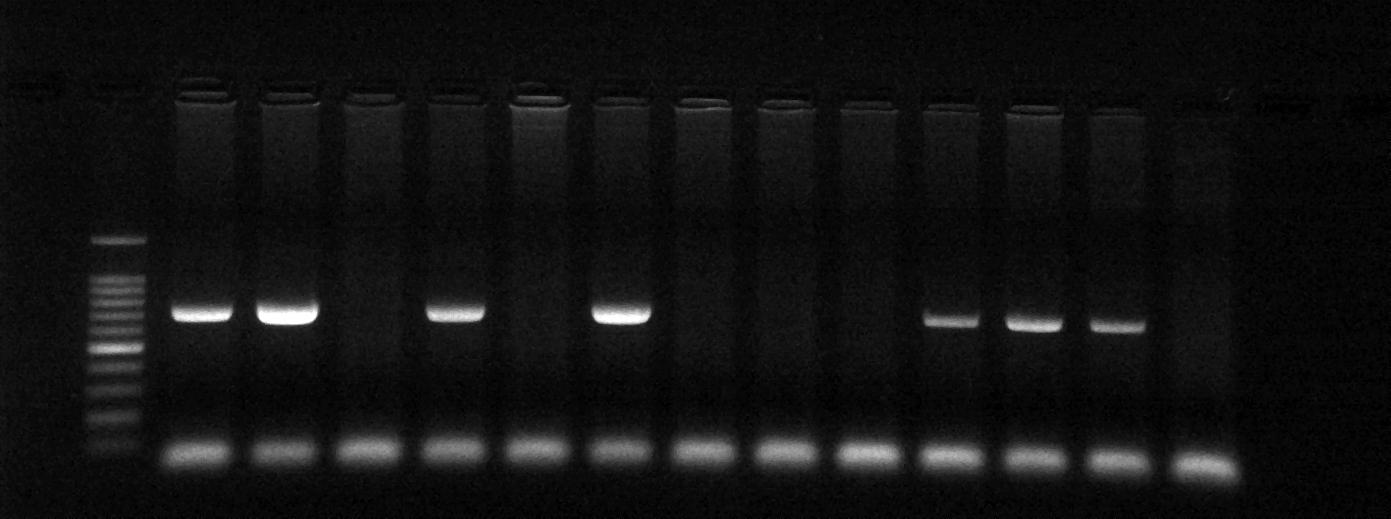

Supplement: Figure 1—figure supplement 1—source data 11. — Relevant parts of the original gel images are presented in Figure 1—figure supplement 1E. [file elife-75863-fig1-figsupp1-data11.jpg]

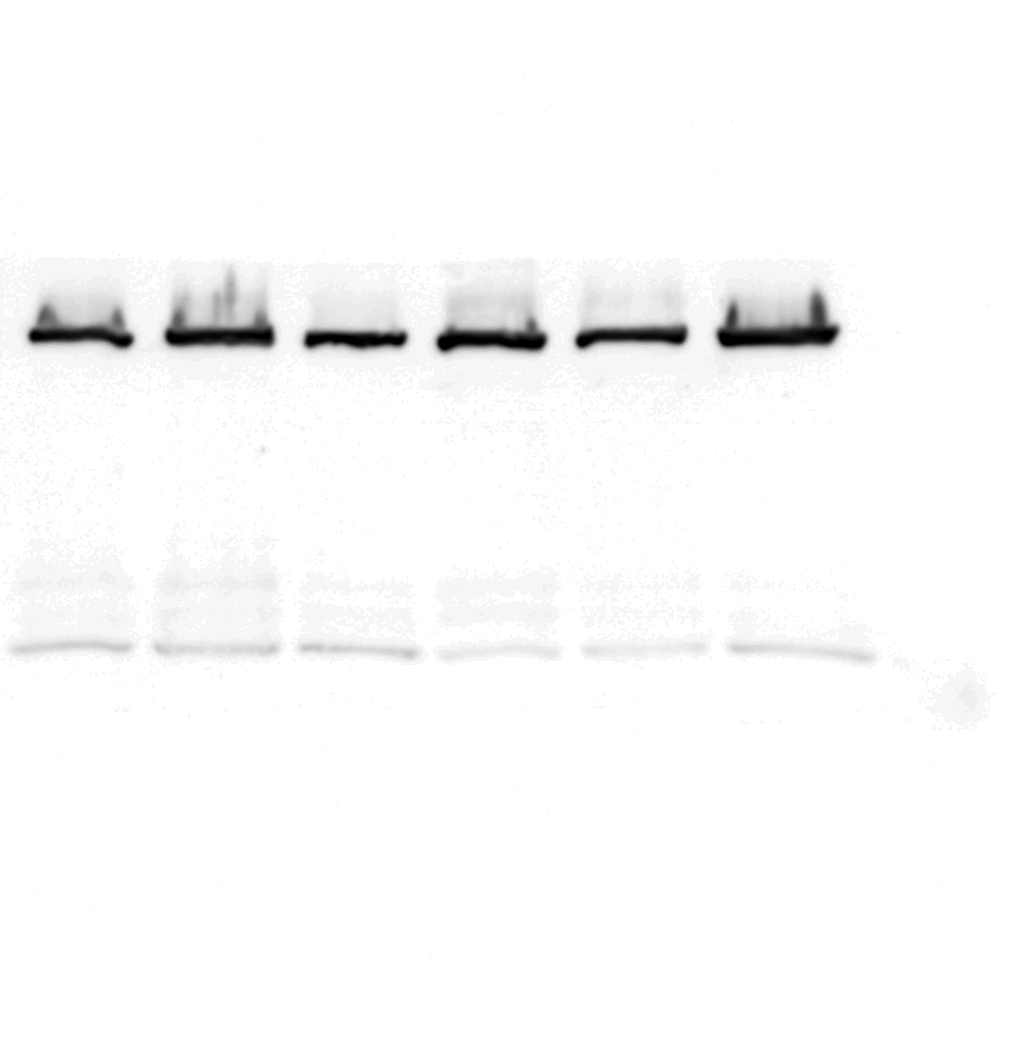

Supplement: Figure 1—figure supplement 1—source data 12. — α-Actinin was used as a normalizing control. Relevant parts of the original gel images are presented in Figure 1—figure supplement 1F. [file elife-75863-fig1-figsupp1-data12.jpg]

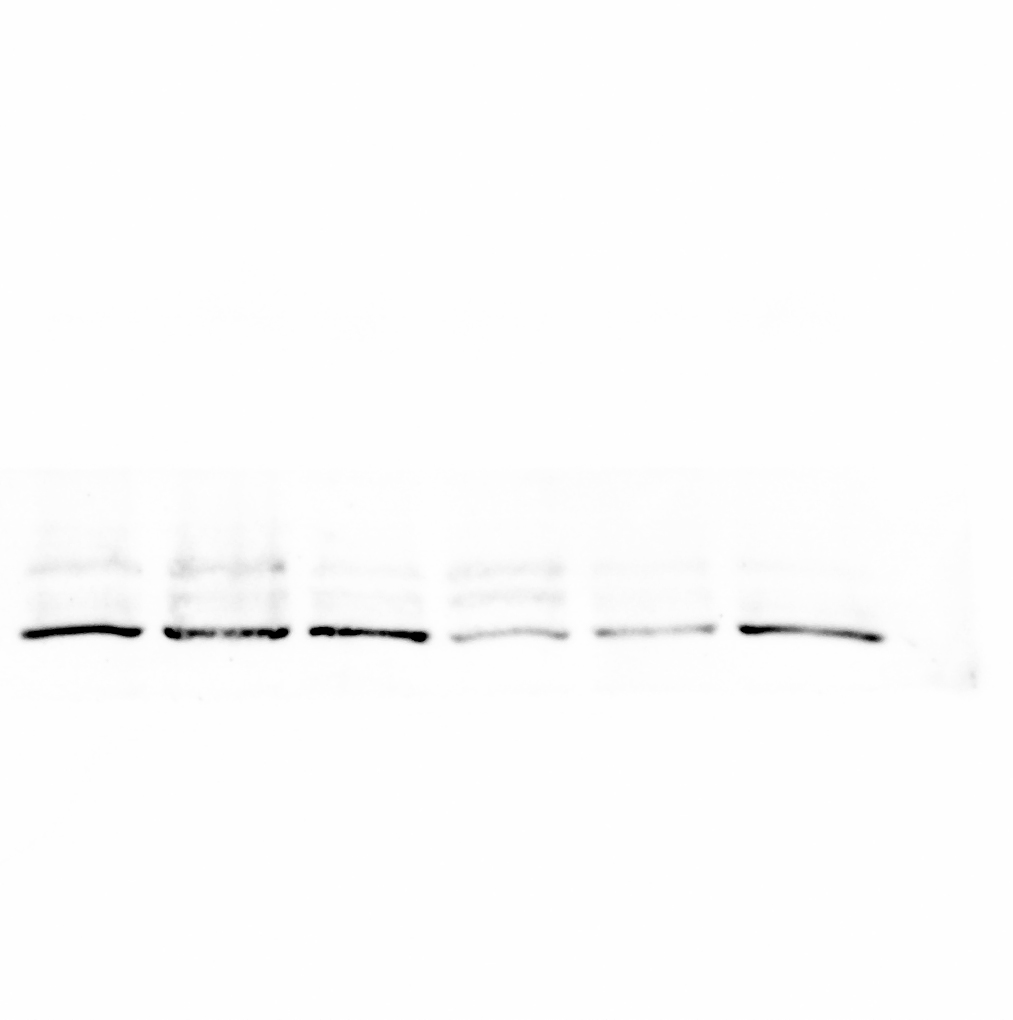

Supplement: Figure 1—figure supplement 1—source data 13. [file elife-75863-fig1-figsupp1-data13.jpg]

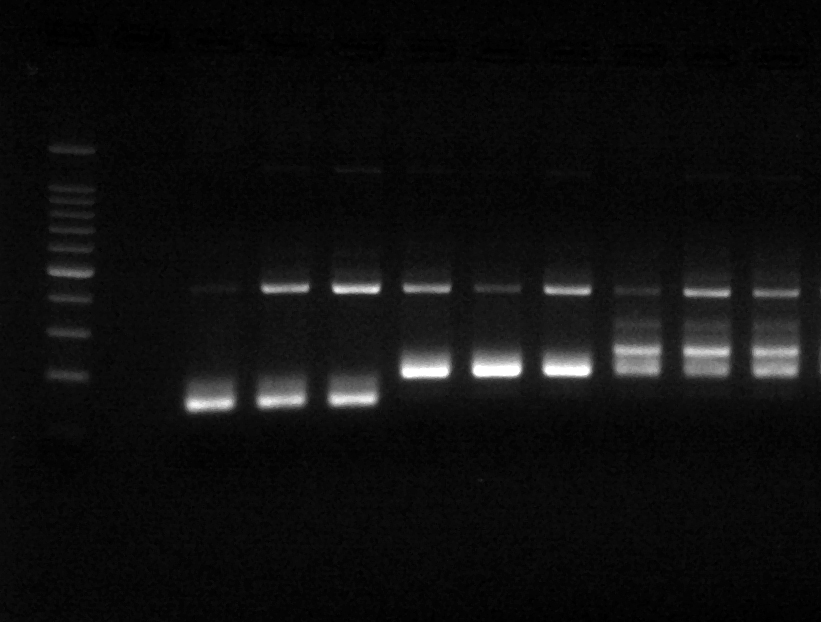

Supplement: Figure 2—source data 1. — In samples originated from Cre- animals, the floxed exon 4, while in wild-type tamoxifen-fed mice the unmodified exon 4 is demonstrated. Relevant parts of the original gel images are presented in Figure 2B. [file elife-75863-fig2-data1.jpg]
