# Supplementary material for: Septin7 is indispensable for proper skeletal muscle architecture and function
Source: eLife. 2022 Aug 5;11:e75863. doi: 10.7554/eLife.75863 (PMC9355566; doi:10.7554/eLife.75863)
Supplement: Supplementary file 1. — (a) Nucleotide sequences, amplification sites, GenBank accession numbers, amplimer sizes, and PCR reaction conditions for each primer pair are shown. Related to Figure 1, Figure 2, and Figure 1—figure supplement 1. (b) Parameters of voluntary running. Related to Figure 2 and Figure 2—figure supplement 2. 3 months of tamoxifen treatment started 4 weeks after birth (4-month-old). 10-day-long running experiment during tamoxifen treatment. *** shows significant difference from Cre- at p<0.001 from t-test. (c) Parameters of in vitro force measurement. Related to Figure 2 and Figure 2—figure supplement 2. 3-month tamoxifen treatment started 4 weeks after birth (4-month-old). *, **, and *** show significant difference from Cre- at p<0.05, 0.01, and 0.001 from t-test, respectively. #, ##, and ### show significant difference from BL6 at p<0.05, 0.01, and 0.001 from t-test, respectively. Fatigue was calculated as the relative amplitude of the 50th tetanus compared to the amplitude of the first tetanus. [file elife-75863-supp1.docx]

**Supplementary File 1a**

| ***Gene*** | *Primer* | *Nucleotide sequence (5’→3’)* | *GenBank  Accession No.* | *Annealing temperature* | *Amplimer size (bp)* |
| --- | --- | --- | --- | --- | --- |
| ***SEPTIN1***  ***Homo sapiens*** | sense | GGGTTTGACTTCACGCTAATG (101-121) | **NM_001365977** | 58 °C | 443 |
|  | antisense | CCAATGACTGGGATGATGTTG (523-543) |  |  |  |
| ***SEPTIN2***  ***Homo sapiens*** | sense | GCGAAGATTCTCATTACC (367–384) | **NM_001008491** | 52 °C | 476 |
|  | antisense | TACCACTGTCAGGCGTAG (825–842) |  |  |  |
| ***SEPTIN3***  ***Homo sapiens*** | sense | TGAATGTTTGCGAATGTTG (4315-4333) | **NM_019106** | 55 °C | 220 |
|  | antisense | GATTGGCTGGGACTGGTA (4517-4534) |  |  |  |
| ***SEPTIN4***  ***Homo sapiens*** | sense | CCGAAAGTCCGTGAAGAA (698-715) | **NM_004574** | 55 °C | 221 |
|  | antisense | TGTCCACAATGGTGAGCC (901-918) |  |  |  |
| ***SEPTIN5***  ***Homo sapiens*** | sense | CTCAACCGAAAGAACATCCAA (513-533) | **NM_002688** | 55°C | 319 |
|  | antisense | TGCCTATAACGGCGAAGG (814-831) |  |  |  |
| ***SEPTIN6***  ***Homo sapiens*** | sense | TTCACGCTTAAACAACCA (3403-3420) | [**NM_**](http://www.ncbi.nlm.nih.gov/entrez/viewer.fcgi?db=nucleotide&val=45383308)**145799** | 52°C | 407 |
|  | antisense | CCTCGTATCCAGGAATGTA (3791-3809) |  |  |  |
| ***SEPTIN7*** | sense | CTTATTGCCAAAGCAGAC (798-815) | **NM_001788** | 50°C | 434 |
| ***Homo sapiens*** | antisense | AGAGGGCTCTTAGTCAGC (1214-1231) |  |  |  |
| ***SEPTIN8*** | sense | TGCCTCTACTTCATCACGC (559-577) | **NM_001098811** | 56 °C | 493 |
| ***Homo sapiens*** | antisense | TGTCACCATCGCTGTCCT (1034-1051) |  |  |  |
| ***SEPTIN9*** | sense | AGGGCACAGATGACCAAAG (3043-3061) | [**NM_001113495.1**](https://www.ncbi.nlm.nih.gov/entrez/viewer.fcgi?db=nucleotide&id=164698501) | 56°C | 287 |
| ***Homo sapiens*** | antisense | GGCAAGTCGGCAAAGTAAA (3311-3329) |  |  |  |
| ***SEPTIN10*** | sense | CTGGCAACAGGCAGCAAC (1449-1466) | [**NM_001321509.2**](https://www.ncbi.nlm.nih.gov/entrez/viewer.fcgi?db=nucleotide&id=1676441741) | 56°C | 366 |
| ***Homo sapiens*** | antisense | AGCAAAGGTGTCGGGTGG (1797-1814) |  |  |  |
| ***SEPTIN11*** | sense | AGGATGATGGGCTTTCTA (2449-2466) | **NM_001306147** | 50 °C | 279 |
| ***Homo sapiens*** | antisense | CTTACGGGTCATGTTGTG (2710-2727) |  |  |  |
| ***SEPTIN12*** | sense | ATGTGGTGCCCGTGATTG (513–530) | **NM_001154458** | 56 °C | 324 |
| ***Homo sapiens*** | antisense | GGAGGTGGGAGCGGATAA (819–836) |  |  |  |
| ***SEPTIN14*** | sense | GAAGAAACTGCTGCTCAA (786-803) | **XM_011515373** | 50 °C | 488 |
| ***Homo sapiens*** | antisense | TTCCTTATCTCCTCCTGT (1256-1273) |  |  |  |
| ***Septin1*** | sense | CACGGCACAAACTCTGACC (222-240) | **NM_017461** | 56°C | 500 |
| ***Mus musculus*** | antisense | CAACGACTGCGAAAGGGA (704-721) |  |  |  |
| ***Septin2*** | sense | GAACAGGCGTCACATCA (550-566) | **NM_001159719** | 50 °C | 352 |
| ***Mus musculus*** | antisense | AACCTTCTTGCCTTTGG (885-901) |  |  |  |
| ***Septin3*** | sense | CCACTGCTGCCTCTACT (776-792) | **NM_001358836** | 52 °C | 231 |
| ***Mus musculus*** | antisense | TTGTCCTCCAAATCCTC (990-1006) |  |  |  |
| ***Septin4*** | sense | GCAAACCGTGGAGATTA (654-670) | **NM_011129** | 52 °C | 325 |
| ***Mus musculus*** | antisense | CGCCTTAGCCAAGATAG (962-978) |  |  |  |
| ***Septin5*** | sense | CGCAAGTCCGTCAAGAAA (194-211) | **NM_213614** | 55 °C | 268 |
| ***Mus musculus*** | antisense | TGGGCTTCCAACATTCAG (444-461) |  |  |  |
| ***Septin6*** | sense | ACATCATTCCCGTTATTGC (820-838) | **NM_001177324** | 52°C | 221 |
| ***Mus musculus*** | antisense | CATCATCTTGTTGCCTATCTT (1020-1040) |  |  |  |
| ***Septin7*** | sense | CCTTGAGGGCTATGTGGG (278-295) | **NM_009859** | 56°C | 250 |
| ***Mus musculus*** | antisense | CAGCAGCAACTGAACACCAC (508-527) |  |  |  |
| ***Septin8*** | sense | CACAGAGGAGGTGAAGGT (1000-1017) | **NM_033144** | 54°C | 471 |
| ***Mus musculus*** | antisense | TTGAAGGCGTTGGTCTC (1454-1470) |  |  |  |
| ***Septin9*** | sense | AACATTGTCCCAGTCATCG (1400-1418) | **NM_001113486** | 54°C | 381 |
| ***Mus musculus*** | antisense | GCGTTTCACTCGGTAGG (1764-1780) |  |  |  |
| ***Septin10*** | sense | GACACGACCTCCAAGAT (1088-1104) | **NM_001024910** | 52°C | 349 |
| ***Mus musculus*** | antisense | GACGCTCACCATAGAAC (1420-1436) |  |  |  |
| ***Septin11*** | sense | CCTGTGCGTGGGTGAGA (287-303) | **NM_001310669** | 55 °C | 433 |
| ***Mus musculus*** | antisense | GATGGTGTCGGCTTTCG (703-719) |  |  |  |
| ***Septin12*** | sense | AGTCCAAAGTATGGCAGTC (344-362) | **NM_027669** | 52°C | 105 |
| ***Mus musculus*** | antisense | GCTTCAATCCCTTCTCC (432-448) |  |  |  |
| ***Septin14*** | sense | TCCACTCTTGGGCATTT (160-176) | **NM_028826** | 50 °C | 290 |
| ***Mus musculus*** | antisense | CTGGCTTCCTTGTTTATCT (431-449) |  |  |  |
| ***HSA-MCM*** | sense | GCATGGTGGAGATCTTTGA |  | 58 °C | 717 |
| ***Mus musculus*** | antisense | CGACCGGCAAACGGACAGAAGC |  |  |  |
| ***Septin7*** | sense1 | CTTTGCACATATGACTAAGC |  | 58 °C | 151 WT |
| ***WT-loxP-KD*** | sense2 | GCTTCTTTTATGTAATCCAGG |  |  | 197 Flox |
| ***Mus musculus*** | antisense | GGTATAGGGGACTTTGGGG |  |  | 256 KD |
| ***Gapdh*** | sense | AAGGTCGGAGTCAACGGATTTGG(99-121) | **NM_001289726.1** | 58 °C | 322 |
| ***Mus musculus*** | antisense | AATGAGCCCCAGCCTTCTCCAT (399-420) |  |  |  |

**Supplementary File 1b**

| **Parameters of running** | **BL6**  **n=3** | **Cre-**  **n=2** | **Cre+**  **n=4** |
| --- | --- | --- | --- |
| **Distance (km/day)** | 2.46±0.19 | 2.34±0.06 | 0.83±0.02*** |
| **Duration (min/day)** | 343.3±15.2 | 317.1±11.1 | 264.4±4.2*** |
| **Average speed (m/min)** | 7.27±0.44 | 7.30±0.32 | 4.01±0.13*** |
| **Maximal speed (m/min)** | 14.04±0.56 | 15.71±0.62 | 10.31±0.20*** |

***p<0.001 from t-test compared to Cre-.

**Supplementary File 1c**

| **Parameters of *in vitro* force** | **Control**  n=3 | | **Cre-**  n=7 | | **Cre+**  n=11 | |
| --- | --- | --- | --- | --- | --- | --- |
|  | **EDL**  n=6 | **soleus**  n=6 | **EDL**  n=14 | **soleus**  n=13 | **EDL**  n=21 | **soleus**  n=20 |
| **Muscle weight (mg)** | 10.4±0.5 | 12.2±0.4 | 12.8±0.6^#^ | 12.3±0.5 | 10.9±0.2*** | 13.6±0.5 |
| **CSA (mm^2^)** | 1.00±0.06 | 0.66±0.05 | 0.76±0.05^#^ | 0.57±0.06 | 0.64±0.04 | 0.68±0.04 |
| **Twitch** | | | | | | |
| **Peak force (mN)** | 2.27±0.08 | 2.42±0.17 | 2.19±0.10 | 2.28±0.12 | 1.85±0.09^#,^** | 1.63±0.13^##,^** |
| **Normalized force (mN/mm^2^)** | 2.30±0.14 | 3.81±0.47 | 3.06±0.26 | 4.54±0.42 | 3.15±0.27 | 2.51±0.23^#,^*** |
| **TTP (ms)** | 33.6±0.8 | 95.7±0.9 | 33.0±1.0 | 81.1±2.3^###^ | 35.3±1.2 | 68.7±2.3^###,^** |
| **HRT (ms)** | 25.9±0.8 | 84.7±5.6 | 26.5±0.7 | 70.8±5.1 | 26.5±0.7 | 58.7±2.7^###,^* |
| **Duration (ms)** | 146.2±14.1 | 310.0±12.3 | 153.8±13.0 | 295.6±22.4 | 129.5±6.5 | 278.7±11.9 |
| **Tetanus** | | | | | | |
| **Peak force (mN)** | 11.24±0.39 | 13.09±0.75 | 10.50±0.26 | 11.45±0.30 | 8.76±0.25^###,^*** | 8.42±0.45^###,^*** |
| **Normalized force (mN/mm^2^)** | 11.22±0.49 | 20.50±2.48 | 14.30±0.89^#^ | 22.43±2.06 | 14.67±1.00 | 12.76±0.98^##,^*** |
| **TTP (ms)** | 165.1±5.1 | 510.1±1.7 | 174.6±8.5 | 513.0±3.0 | 151.6±5.6* | 492.5±13.8 |
| **HRT (ms)** | 78.3±7.1 | 131.4±3.1 | 74.2±11.0 | 108.3±2.7^###^ | 91.2±5.6 | 101.5±14.2 |
| **Duration (ms)** | 335.6±8.9 | 768.2±11.3 | 331.8±5.9 | 740.4±9.9^##^ | 311.4±5.3^#,^* | 691.9±5.9^##,^*** |
| **Fatigue at 50^th^ tetanus (%)** | 32.1±1.2 | 23.9±4.6 | 25.8±1.6^#^ | 23.6±1.7 | 33.3±2.1* | 34.0±1.8^#,^*** |

^#^p<0.05, ^##^p<0.01, ^###^p<0.001 from t-test compared to Control.

*p<0.05, **p<0.01,***p<0.001 from t-test compared to Cre-.
