# Supplementary material for: Septin7 is indispensable for proper skeletal muscle architecture and function
Source: eLife. 2022 Aug 5;11:e75863. doi: 10.7554/eLife.75863 (PMC9355566; doi:10.7554/eLife.75863)
Supplement: Transparent reporting form [file elife-75863-transrepform1.pdf]

eLIFE
1st Floor
24 Hills Road
Cambridge CB2 1JP, UK
P 01223 855340
W elifesciences.org
T @elife

eLife's transparent reporting form

We encourage authors to provide detailed information *within their submission* to facilitate the interpretation and replication of experiments. Authors can upload supporting documentation to indicate the use of appropriate reporting guidelines for health-related research (see EQUATOR Network), life science research (see the BioSharing Information Resource), or the ARRIVE guidelines for reporting work involving animal research. Where applicable, authors should refer to any relevant reporting standards documents in this form.

If you have any questions, please consult our Journal Policies and/or contact us: editorial@elifesciences.org.

Sample-size estimation

- You should state whether an appropriate sample size was computed when the study was being designed
- You should state the statistical method of sample size computation and any required assumptions
- If no explicit power analysis was used, you should describe how you decided what sample (replicate) size (number) to use

Please outline where this information can be found within the submission (e.g., sections or figure legends), or explain why this information doesn't apply to your submission:

We used G*Power software (http://www.gpower.hhu.de) to calculate the optimal sample size for our experiments.

Replicates

- You should report how often each experiment was performed
- You should include a definition of biological versus technical replication
- The data obtained should be provided and sufficient information should be provided to indicate the number of independent biological and/or technical replicates
- If you encountered any outliers, you should describe how these were handled
- Criteria for exclusion/inclusion of data should be clearly stated
- High-throughput sequence data should be uploaded before submission, with a private link for reviewers provided (these are available from both GEO and ArrayExpress)

Please outline where this information can be found within the submission (e.g., sections or figure legends), or explain why this information doesn't apply to your submission:

All information regarding the number of each experiments, individual biological samples and technical replicates can be found in the appropriate figure legends.

Statistical reporting

- Statistical analysis methods should be described and justified
- Raw data should be presented in figures whenever informative to do so (typically when N per group is less than 10)
- For each experiment, you should identify the statistical tests used, exact values of N, definitions of center, methods of multiple test correction, and dispersion and precision measures (e.g., mean, median, SD, SEM, confidence intervals; and, for the major substantive results, a measure of effect size (e.g., Pearson's r, Cohen's d)
- Report exact p-values wherever possible alongside the summary statistics and 95% confidence intervals. These should be reported for all key questions and not only when the p-value is less than 0.05.

Please outline where this information can be found within the submission (e.g., sections or figure legends), or explain why this information doesn't apply to your submission:

Raw data are presented in the appropiate Figure panels, where N is lower than 150. In other cases, pooled data are expressed as mean±standard error of the mean (SEM). The differences between control mice and animals on tamoxifen diet were assessed using one-way analysis of variance (ANOVA) and all pairwise Bonferroni's multiple comparison method using the statistical program Prism (GraphPad Software, San Diego, CA, USA). In the molecular biological methods (e.g. PCR, denzitometry of Western blot signals, proliferation assay, data analysis from EM images), T-test was used to test the significance and a p value of less than 0.05 was considered statistically significant.
Significant differences between the appropriate data are labelled in Figures and given in the Figure legends.

(For large datasets, or papers with a very large number of statistical tests, you may upload a single table file with tests, Ns, etc., with reference to sections in the manuscript.)

Group allocation

- Indicate how samples were allocated into experimental groups (in the case of clinical studies, please specify allocation to treatment method); if randomization was used, please also state if restricted randomization was applied
- Indicate if masking was used during group allocation, data collection and/or data analysis

Please outline where this information can be found within the submission (e.g., sections or figure legends), or explain why this information doesn't apply to your submission:

Samples originated from control cultured cells or mice, and from Septin-7 modified cells and animals were used for the appropriate experimental groups. Human samples from *m. quadriceps femoris* of human patients going through amputation were taken and used in this study. We did not use masking during group allocation and data collection/analysis.

Additional data files ("source data")

- We encourage you to upload relevant additional data files, such as numerical data that are represented as a graph in a figure, or as a summary table
- Where provided, these should be in the most useful format, and they can be uploaded as "Source data" files linked to a main figure or table
- Include model definition files including the full list of parameters used
- Include code used for data analysis (e.g., R, MatLab)
- Avoid stating that data files are "available upon request"

eLife Sciences Publications, Ltd is a limited liability non-profit non-stock corporation incorporated in the State of Delaware, USA, with company number 5030732, and is registered in the UK with company number FC030576 and branch number BR015634 at the address 1st Floor, 24 Hills Road, Cambridge CB2 1JP | August 2014

Please indicate the figures or tables for which source data files have been provided:

Key resources table containing reagents used in the different experimental procedures, model systems, devices and softwares are listed in the Supplementary information file. The data that support the findings will be available in the data repository of University of Debrecen at https://adattar.unideb.hu/ following the date of publication.
